# Integrating GWAS and Gene Expression Analysis Identifies Candidate Genes for Root Morphology Traits in Maize at the Seedling Stage

**DOI:** 10.3390/genes10100773

**Published:** 2019-10-02

**Authors:** Houmiao Wang, Jie Wei, Pengcheng Li, Yunyun Wang, Zhenzhen Ge, Jiayi Qian, Yingying Fan, Jinran Ni, Yang Xu, Zefeng Yang, Chenwu Xu

**Affiliations:** 1 Jiangsu Key Laboratory of Crop Genetics and Physiology/ Key Laboratory of Plant Functional Genomics of the Ministry of Education/ Jiangsu Key Laboratory of Crop Genomics and Molecular Breeding, Agricultural College of Yangzhou University, Yangzhou 225009, China; houmiaowang@yzu.edu.cn (H.W.); MX120170578@yzu.edu.cn (J.W.); pcli@yzu.edu.cn (P.L.); DX120180078@yzu.edu.cn (Y.W.); MX120170587@yzu.edu.cn (Z.G.); M160588@yzu.edu.cn (J.Q.); MX120170589@yzu.edu.cn (Y.F.); MX120170575@yzu.edu.cn (J.N.); yangx@yzu.edu.cn (Y.X.); zfyang@yzu.edu.cn (Z.Y.); 2 Jiangsu Co-Innovation Center for Modern Production Technology of Grain Crops, Yangzhou University, Yangzhou 225009, China; 3 Joint International Research Laboratory of Agriculture and Agri-Product Safety of Ministry of Education of China, Yangzhou University, Yangzhou 225009, China

**Keywords:** root traits, maize, quantitative trait locus, GWAS, RNA-seq, candidate genes

## Abstract

Root system plays an essential role in water and nutrient acquisition in plants. Understanding the genetic basis of root development will be beneficial for breeding new cultivars with efficient root system to enhance resource use efficiency in maize. Here, the natural variation of 13 root and 3 shoot traits was evaluated in 297 maize inbred lines and genome-wide association mapping was conducted to identify SNPs associated with target traits. All measured traits exhibited 2.02- to 21.36-fold variations. A total of 34 quantitative trait loci (QTLs) were detected for 13 traits, and each individual QTL explained 5.7% to 15.9% of the phenotypic variance. Three pleiotropic QTLs involving five root traits were identified; SNP_2_104416607 was associated with lateral root length (LRL), root surface area (RA), root length between 0 and 0.5mm in diameter (RL005), and total root length (TRL); SNP_2_184016997 was associated with RV and RA, and SNP_4_168917747 was associated with LRL, RA and TRL. The expression levels of candidate genes in root QTLs were evaluated by RNA-seq among three long-root lines and three short-root lines. A total of five genes that showed differential expression between the long- and short-root lines were identified as promising candidate genes for the target traits. These QTLs and the potential candidate genes are important source data to understand root development and genetic improvement of root traits in maize.

## 1. Introduction

Over the next 30 years, the global human population is expected to grow by 25% and reach 10 billion, but the current pace of yield increase for major crops is insufficient to meet future demand [1]. Water shortages, nutrient deficiency, and lodging caused by extreme weather can severely reduce crop productivity and threaten global food security [2]. Root systems provide mechanical support, uptake water and nutrients, and interact with rhizosphere microbes and are vital for plant growth and adaptation [3]. After germination, root systems continue to expand in size to become structurally and functionally complex and sometimes may be bigger than above-ground parts [4]. Root systems have developmental plasticity and are capable of altering root branching, root angle, and root growth rates to adapt to adverse soil conditions [5]. The root system architecture is instrumental for superior productivity, but it remains a largely untapped resource in plant breeding programs [6].

The complex maize root system comprises five main types of roots: primary, seminal, crown, brace, and lateral roots. Embryonic roots, including primary and seminal roots, are important for maize early development. The relationship between early root development and crop productivity and nutrient use efficiency has been revealed by genetic and phenotypic studies [7,8,9]. Crown and brace roots have a major role in anchorage and soil resource acquisition in later growth [10]. Lateral roots are a major determinant of root architecture. Maize genotypes with reduced lateral root branching showed high drought tolerance, whereas long lateral roots were optimal for nitrate acquisition, and short lateral roots were optimal for phosphorus acquisition [11]. Root architecture involves complex, multicomponent, and interconnected traits, and optimal root system architecture has a major impact on soil resource usage and yield productivity [12].

Although root traits can play key roles in enhancing resource use efficiency and plant adaptation, root traits have seldom been considered as selection criteria in maize breeding programs, mainly because only a few root genes have been identified. The monogenes *rtcs*, *rtcl*, *rum1*, *Bige1*, *rth1*, *rth3*, *rth5*, and *rth6* have been cloned in maize [6]. In rice, a large number of genes related to root development have been identified (http://www.ricedata.cn/ontology/ontology.aspx?ta=GO:0048364). For example, *ARL1*, *CRL4*, *OsPIN1*, *OsIAA23*, and *OsCAND1*, which are involved in auxin signaling, play essential roles in crown root development [13]. Several studies showed that genes involved in sugar metabolism (*OsGNA1*, *OsMOGS*, and *OsDGL1*) and cell wall biosynthesis (*OsGLU3* and *OsEXPA8*) play important roles in root elongation [14]. Recently, two transcription factors *OsMADS25* and *OsSPL3* were found to control primary root length, lateral root density, and crown root development [13,15]. Most of these genes have not been targeted for breeding improvement and only two quantitative trait loci (QTLs), deeper rooting 1 (*DRO1*) and phosphorus starvation tolerance 1 (*PSTOL1*), have been used to improve drought and low phosphorus tolerance in rice [16,17]. In maize, although a large number of root QTLs have been mapped, no QTLs have been cloned.

Because maize displays abundant genetic diversity and rapid linkage disequilibrium (LD) decay, it has become the model crop for association analysis, and several genes associated with complex traits have been identified [18,19,20]. This approach has been successfully used to pinpoint root genes. By integrating genome-wide association studies (GWAS) and gene expression analyses, Meijón et al. (2013) identified a new F-box gene *KUK* that was associated with root cell length in Arabidopsis [21]. A brassinosteroid signaling kinase BSK3 that modulated root elongation under mild nitrogen deficiency also was identified by GWAS [22]. In rice, GWAS was conducted for 21 root traits under normal and drought stress, and 143 significant associations were identified, including 11 reported root-related genes [23]. Additionally, new causal genes *Nal1* and *OsJAZ1* were validated by sequence variation, expression, and transgenic experiments [23]. Forty-four and 97 QTL candidate genes for root length and root thickness were identified by GWAS, and linkage mapping, genomic, transcriptomic, and haplotype data were obtained; five of the root length QTL candidates were validated by T-DNA insertional mutation [24]. In maize, GWAS was conducted for 22 root traits in 384 inbred lines, and 268 marker–trait associations were identified. GRMZM2G153722, which is located on chromosome 4, contained nine significant markers and was the most likely candidate gene; however, no further validation was conducted [25]. Therefore, many loci that affect root development remain unknown in maize.

Another reason for the poor consideration of roots in breeding programs is that root traits are difficult to evaluate in the field. Roots are hidden in soil, and their exceptional degree of plasticity to changing soil conditions makes them inaccessible to direct genetic analyses [6]. Although many new approaches have been developed to evaluate root traits in both laboratory and field conditions [26], paper roll under hydroponic conditions was found to be the best system to access root phenotypes at the seedling stage. These indoor culture systems allow high-throughput and accurate evaluation of root traits for large-scale genetic screens [27]. In this study, a panel of 297 maize inbred lines [28,29] was used to conduct GWAS to investigate maize root architecture at the seedling stage. RNA-seq was conducted with three long-root lines and three short-root lines to obtain the expression levels of candidate genes. The objectives of this study were (i) to study phenotypic variation of 16 root and shoot traits within a maize association panel, (ii) to identify significant SNPs associated with root traits, and (iii) to employ GWAS and RNA-seq data to explore potential candidate genes for root development.

## 2. Materials and Methods

### 2.1. Plant Materials and Growth Conditions

A total of 297 maize inbred lines [28,29] were evaluated for root traits and used for GWAS. Root traits were measured in a paper roll system as described previously [27,29]. Briefly, seeds of similar size were selected for germination and surface sterilized with 10% H_2_O_2_ solution for 20 min. The sterilized seeds were washed twice with distilled water, soaked in saturated CaSO_4_ for 6 h, then germinated in the dark on moist filter paper at 28 °C and 80% relative humidity. After 2 days, eight germinated seeds from each of the 297 genotypes were vertically rolled in two-layer brown germination roll paper (Anchor Paper Company, St Paul, MN, USA). The germination paper rolls were placed in 39.5 × 29.5 × 22.5 cm black incubators containing 7.5 L nutrient solution, as shown in Table S1. The nutrient solution was renewed every 3 days. Plants were grown in a greenhouse located on the campus of Yangzhou University in September to October in 2018. The experiment was conducted in a completely randomized design with two replicates.

### 2.2. Plant Phenotyping and Data Analysis

Seedlings were harvested at 14 days after germination, and the roots were separated from the shoots and stored at 4 °C. Roots were also scanned to produce high resolution images that were analyzed using WinRHIZO software (Pro 2004b, Quebec, Canada). To determine root and shoot dry weights, roots and shoots were collected separately and dried at 105 °C for 30 min and then at 55 °C until a constant weight was achieved. In total, 16 traits including 13 root traits and 3 shoot traits were measured (Table 1). Statistical analysis was performed using the R software package (R Development Core Team 2013). Mean values of each line were used for subsequent phenotypic summarization, correlation analysis, principal component analysis, and trait–marker associations. Principal component analyses were performed using the “prcomp” function in R with further visualization performed using the “factoextra” package.

### 2.3. Genotypic Data and Genome-Wide Association Analysis

The panel of 297 inbred lines was genotyped using the genotyping-by-sequencing strategy [28,29]. The *MseI* restriction enzyme was used for library preparation, and GBS was performed on an Illumina platform by Novogene Bioinformatics Institute, Beijing, China. The clean reads from each sample were mapped to the B73 RefGen_V3 reference genome sequence (AGPv3, release 31) by BWA with the settings ‘mem -t 4 -k 32 -M’ [30]; variant calling was performed for all samples using the SAMtools software (V1.3.1) [31]. After quality control (missing rate ≥20%; minor allele frequency ≥0.05) 131,271 SNPs remained for GWAS. Principal component analysis was performed using TASSEL 5.0, and the top five principal components were used to create a population structure matrix to control population structure. Kinship matrices were calculated using the Centered-IBS method in TASSEL 5.0 to estimate genetic relatedness among individuals. We performed GWAS with a mixed linear model (MLM) with principal components and kinship and a general linear model (GLM) with principal components in TASSEL 5.0. Because several SNPs were in LD, the effective numbers of independent SNPs (35,831) was estimated using the GEC software tool (V0.2) [32] with the significant *P*-value threshold set as 2.79 × 10^−5^ (1/independent marker number). Considering the complexity of root traits, a suggestive *P*-value threshold (1 × 10^−4^) was used to avoid ignoring minor effect loci. The decay distance of LD across the whole genome was determined by software PopLDdecay [33]. Based on the average LD decay of 50 kb, significant SNPs within 50 kb were binned into a QTL, and the most significant SNP were selected as the lead SNP.

### 2.4. Candidate Gene Analysis and Transcriptome Sequencing

All the potential candidate genes within 100 kb (50-kb upstream and 50-kb downstream of the lead SNP) of the detected loci were identified. Gene annotation information from maize GDB database (http://www.maizegdb.org) was used to assign functions to the candidate genes. The physical locations of the genes and SNPs were based on the maize B73 RefGen_V3 genome (AGPv3, release 31).

Three long-root lines and three short-root lines were used for the transcriptomic analysis. The six lines were planted in the paper roll system described above. Fourteen days after germination, roots of each line were sampled for total RNA extraction using an RNeasy R Plant Mini kit (Qiagen, Shanghai, China). High quality RNA was used to construct an RNA-seq library for each sample using a TruSeq RNA sample preparation kit (Illumina). Clean data were obtained by removing adapters, low-quality reads, and reads containing poly-N from the raw data. High-quality reads were aligned to the B73 RefGen_V3 reference genome sequence (AGPv3, release 31) using HISAT2 (V2.1.0). FeatureCounts in the Subread package (v1.6.5) was used to count the read numbers mapped to each gene [34]. The FPKM (fragments per kilobase of exon model per million mapped reads) of each gene was calculated based on the length of the gene and read count mapped to it [35]. The R package ‘edgeR’ was used to identify differentially expressed genes. Differentially expressed genes were identified by calculating the log2 fold change (FC) between the genes from the long- and short-root lines. DEGs were selected with |log2 (FC)| > 1 and with statistical significance *P* < 0.05. SNP calling from RNAseq data was performed with ‘the GATK Best Practices workflow for SNP and indel calling on RNAseq data’ [36]. The identified SNPs were annotated using snpEff [37]. The sequencing data were deposited in the NCBI Short Read Archive database with the accession number PRJNA558447. The basic information of the transcriptomic data is provided in Supplementary Dataset 3.

## 3. Results

### 3.1. Phenotypic Analysis of Root Traits at the Seedling Stage

**Figure 1:**
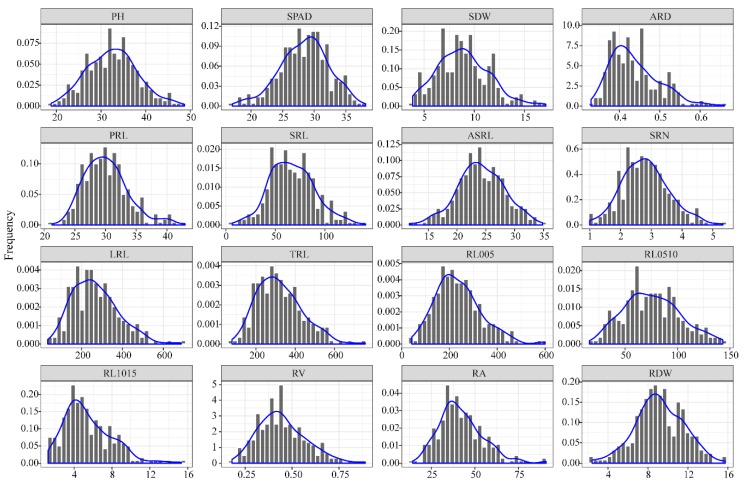
Distribution of root and shoot traits in 297 maize inbred lines.

To dissect the genetic basis of root traits at the maize seedling stage, 297 inbred maize lines were cultured in paper rolls for two weeks. A total of 18 traits, including shoot dry weight (SDW), total root length (TRL), lateral root length (LRL), primary root length (PRL), average root diameter (ARD), root surface area (RA), root volume (RV), and root dry weight (RDW), were measured (Table S1). All the captured traits showed a continuous distribution with a slight left skew (Figure 1). For most of the traits, considerable phenotypic variation was detected among the lines, with coefficients of variation that ranged from 12.01% for PRL to 46.22% for RL1015. PRL varied from 21.27 to 41.98 with an average of 30.12, and RL1015 varied from 1.24 to 15.32 with an average of 5.43. All traits exhibited >2-fold differences and ranged from 2.02 to 21.36 among the lines (Table 2).

Phenotypic correlation analysis showed that most measured traits were closely positively correlated with each other, expect ARD (Figure S1). Strong positive correlations were found for TRL, LRL, RL005, RL0510, RL1015, RV, and RA (*r* = 0.45–0.99). Principal component analyses were conducted for 16 traits, and three major principal components that accounted for more than 70% of the phenotypic variance were detected (Figure S2). The first dimension (Dim1) represented 49.9% of the variability and accounted primarily for nine traits RA, TRL, LRL, RL005, RV, RL0510, SDW, PH, and SRL. Dim2 explained 12.2% of the variation and accounted mainly for ARD (Figure S2). The results are consistent with the phenotypic correlation analysis.

### 3.2. Genome-Wide Association Studies

**Figure 2:**
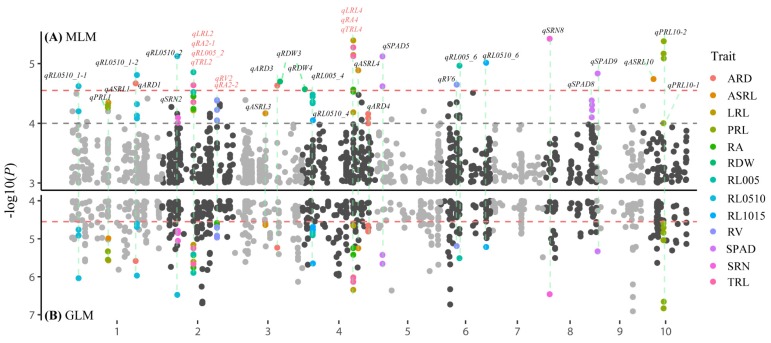
Manhattan plots of mapped single nucleotide polymorphisms (SNP) markers associating with each trait. (**A**) Mixed Linear Model (MLM), (**B**) General Linear Model (GLM). Abbreviations for traits are as follows: ARD, average root diameter; ASRL, average seminal root length; LRL, lateral root length; PRL, primary root length; RA, root surface area; RDW, root dry weight; RL005, root length between 0 and 0.5 mm in diameter; RL0510, root length between 0.5mm and 1mm in diameter; RL1015, root length greater than 1 mm in diameter; RV, root volume; SPAD, leaf chlorophyll concentrations measured by SPAD-502 PLUS chlorophyll (Minolta, Japan); SRN, seminal roots numbers; TRL, total root length.

A set of 131,271 filtered SNPs with minor allele frequencies >0.05 were used for GWAS with GLM and MLM. A total of 355 and 28 marker–trait associations were identified by GLM and MLM, respectively (Supplementary Dataset 1). As expected, MLM was too strict, whereas there were a large number of false positives for GLM because of the population structure. We found that 27 of the 28 significant associations from MLM also were detected by GLM. Some close-by SNPs that showed strong LD could not be considered as separate loci, so the 28 significant associations were clumped into 21 significant QTLs based on an average LD decay of 50 kb (Figure S3). To avoid missing SNPs because of the strict MLM, we defined suggestive QTLs as those with at least one SNP [−log_10_(*P*) > 4] in MLM and two SNPs [−log_10_(*P*) > 4.55] in GLM within 50-kb distances of each other. For example, *qPRL1* was selected as a suggestive QTL because in a 50-kb genomic region, two SNPs were associated with PRL in MLM [−log_10_(*P*) > 4], and the same two SNPs were identified in GLM [−log_10_(*P*) > 4.55] (Table 3; Figure 2). A total of 21 significant QTLs and 13 suggestive QTLs were detected for 13 traits; except SRL, SDW, and PH (Table 3; Figure 2). These QTLs were distributed on all 10 maize chromosomes with the highest number on chromosomes 4 and 8, each of which contained eight QTLs (Figure 2). Individually, these QTLs explained between 5.7% and 15.9% of the phenotypic variance (Table 3). A total of 19 QTLs were detected for seven root length traits, namely, TRL (2), LRL (2), RL005 (3), RL0510 (3), RL1015 (2), PRL (3), and ASRL (4). QTLs for TRL were completely coincident with QTLs for LRL. Among the 19 QTLs, nine explained more than 10% of the phenotypic variation (Table 3). Three QTLs were detected for ARD, and the most significant association was for SNP_1_213315833, which explained 8.5% of the phenotypic variance. We found 3, 2, and 2 QTLs that were associated with RA, RV, and RDW, respectively. The most significant SNP for RA was SNP_2_104416607, which explained 14.65% of the phenotypic variation. For SRN and SPAD, 2 and 3 QTLs were found, respectively. Three pleiotropic QTLs involving five root traits were identified; SNP_2_104416607 was associated with LRL, RA, RL005, and TRL; SNP_2_184016997 was associated with RV and RA; and SNP_4_168917747 was associated with LRL, RA, and TRL (Figure 2).

### 3.3. Determination of Candidate Genes

**Figure 3:**
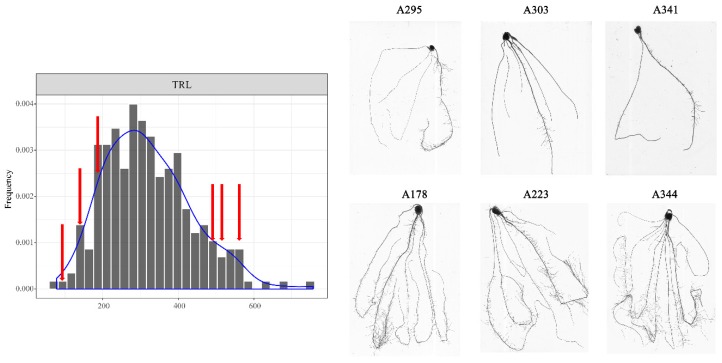
Scanned root images of 3 short-root lines and 3 long-root lines.

**Figure 4:**
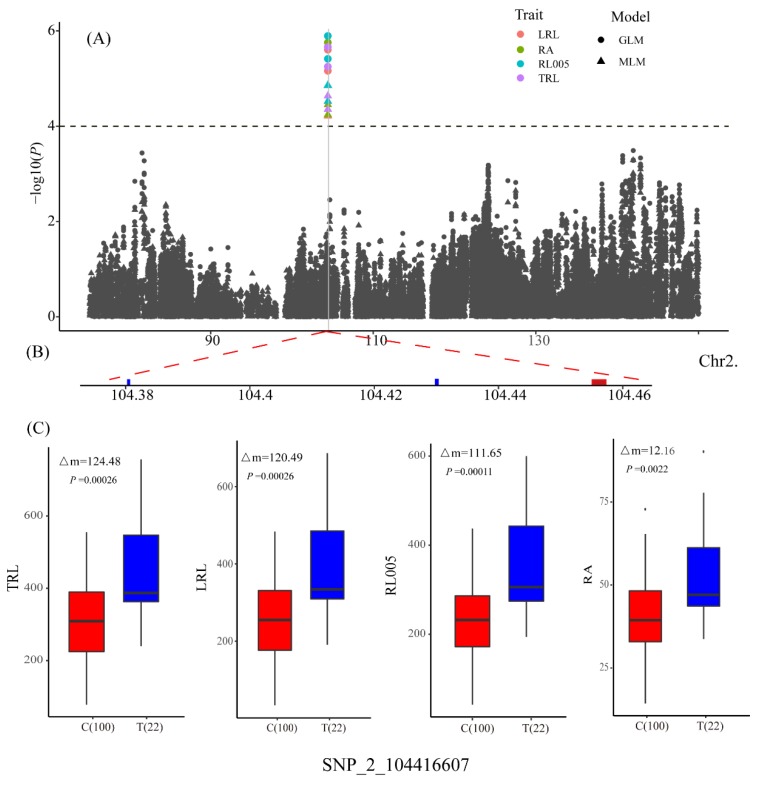
Association analysis of pleiotropic SNP_2_104416607 with LRL, RA, RL005, and TRL. (**A**) Manhattan plots of association analysis, (**B**) Candidate genes around SNP_2_104416607, (**C**) The box shows phenotypic differences between lines carrying different alleles of SNP_2_104416607, the difference of mean (Δm), and the *P*-value are given.

A total of 96 candidate genes were located within the ± 50-kb (Figure S3) genomic regions of the lead SNPs for the identified QTLs (Supplementary Dataset 2). To reduce the number of candidate genes, three long-root lines and three short-root lines were selected from among the 297 inbred lines and performed RNA-seq to evaluate the whole-genome gene expression levels (Figure 3). A total of 4458 DEGs were identified between long-root lines and short-root lines (Supplementary Dataset 3). Within loci identified by GWAS, we detected 78 genes that were not expressed in all lines and 5 genes were differential expressed between the long- and short-root lines (Table 4). Further, the variants were identified from the RNA-seq data, and a total of 805,328 SNPs were detected in the whole genome, including 1083 SNPs located in 42 candidate genes for the identified root QTLs (Supplementary Dataset 4 and 5). A total of 84 SNPs located in UTR (untranslated regions) and CDS (coding DNA sequence) were identified for five DEGs. The pleiotropic marker SNP_2_104416607 on chromosome 2 was significantly associated with four root traits (LRL, RA, RL005, and TRL), and the increased allele (T) increased the phenotype by 15% relative to the alternative allele (Figure 4). Three genes (GRMZM2G112828, GRMZM2G112838, and GRMZM2G397965) were detected in the 104,366,607–104,466,607 bp region around SNP_2_104416607 on chromosome 2. The transcriptome analysis showed that GRMZM2G112828 and GRMZM2G112838 were not expressed, and GRMZM2G397965 was up-regulated with log_2_(FC) = 2.78 in the long-root lines compared with the short-root lines. Eight SNPs, including three non-synonymous mutations and two mutations in untranslated regions, may affect expression level of GRMZM2G397965 (Table 4). SNP_3_ 147397047 was found to be significantly associated with ARD, and four genes (AC184770.3_FG001, GRMZM2G138258, GRMZM2G138338 and GRMZM2G138342) were identified in the 147,347,047–147,447,047 bp region around SNP_3_ 147397047 on chromosome 3. GRMZM2G138338 was up-regulated with log_2_(FC) = 1.38 in the long-root lines (Table 4). Eight non-synonymous mutations were detected in this gene. Two DEGs, GRMZM2G174797 and GRMZM2G476902, located in *qASRL4*, GRMZM2G174797 was up-regulated with log_2_(FC) = 1.44, while GRMZM2G476902 was down-regulated [(log_2_(FC) = 1.44] in the long-root lines. The candidate gene GRMZM2G031528 for *qRDW3* is predicted to code for a heavy metal transport/detoxification superfamily protein. It contained one non-synonymous mutation and eight UTR variants. By integrating the results of the GWAS and transcriptomic analyses, we detected five potential genes for the root traits.

## 4. Discussion

Roots anchor plants in the soil and absorb nutrients and water from soil environments. However, phenotyping root traits is difficult, especially for a large plant, like maize. Several methods for root phenotyping in the laboratory have been developed; hydroponics and paper roll culture are the most commonly used methods for root research because they allow rapid, accurate, and high-throughput screening of root characteristics in a large number of genotypes at the seedling stage. Almost half of all root genetics research in maize, barley, and wheat used this approach [6,38]. Here, we grew 297 inbred maize lines under standardized conditions in paper rolls to get accurate phenotypes at the seedling stage, including root length traits (SRL, TRL, and LRL) that are important for nutrient uptake efficiency and grain yield [7]. Understanding the natural variation of root traits and identifying SNPs associated with the variation is a prerequisite for the improvement of maize roots by molecular breeding technology. Abundant phenotypic diversity is essential for the success of genetic studies. In this study, root traits showed 2.00–21.36-fold differences in 297 inbred lines (Table 2), which is similar to that of other natural populations [25,39]. In previous studies, more extensive phenotypic variation was observed. In a panel of 30 maize inbred lines, the seminal root number ranged from 0 to 10 [40]. Root traits in maize landraces and teosinte were highly variable in the number of nodal and seminal roots, making them a valuable resource for identifying natural variations to improve root traits in maize [41]. Currently, the only cloned root QTLs in crop species were found in rice, namely genes *DRO1* and *PSTOL1*. *DRO1* enhances root gravitropism to increase the root growth angle, which maintained high yield performance under drought conditions; the functional variation of *DRO1* was found in a drought-tolerant cultivar ‘Kinandang Patong’ from the Philippines [16]. *PSTOL1* acts as an enhancer of early root growth and promotes root growth under both high and low phosphorus conditions; the functional variation was found in the low phosphorus-tolerant *aus*-type ‘Kasalath’ [17]. These two genes were identified in landrace germplasm rather than elite breeding lines [42]. During domestication, maize and soybean genes associated with root system architecture were both under indirect selection [43,44], suggesting that both the inbred lines and the wild ancestor retained a lot of variation for root traits. In this study, maize inbred lines with extensive phenotypic variation allowed the discovery of significant SNPs underlying root traits.

GWAS was a powerful tool to detect natural variations for complex traits, but complex population structure and ancestral relatedness in natural population reduced the mapping power. Even though several statistical methods have also been developed to increase statistical power, there are still many restrictions [45]. For example, there were a large number of false positives for GLM, but MLM can lead to false negatives by overcompensating for population structure and kinship. Only four SNPs were detected for 22 root traits by MLM using 135,311 SNP markers in 384 inbred lines [25]. Many studies have used both approaches as well as suggestive and significant association level to balance the false positives and false negatives in root studies [23,46]. Here, 21 significant and 13 suggestive QTLs were detected by integrating the results of GLM and MLM. GWAS has been used successfully to identify thousands of root-associated loci in Arabidopsis, rice, wheat, barley, and maize [21,22,23,24,25], and some important genes and variations have been detected. However, these significant loci explained only a few percent of the phenotypic diversity. For example in barely, the detected root QTLs cumulatively explained 12.1%–48.1% of the phenotypic variance [46]. In this study, we detected from two to four QTLs for each trait that jointly explained less than 40% of the total phenotypic variation (Table 3), so a large proportion of the heritability was missed. We propose two possible reasons for this. One, root traits are controlled by numerous small-effect QTLs, which is why the phenotypic variation explained by each QTL ranged from 5.7% to 15.9% in our study. Even in bi-parental populations most root QTLs explained less than 10% of the phenotypic variation [7,47]. The other reason may be that the presence of rare variants reduced the statistical power of GWAS. Multi-parent populations such as NAM and MAGIC may make powerful tools for unraveling the molecular basis of maize root development. Here, a total of 34 QTLs were detected for 12 root traits and SPAD value, and 96 candidate genes within 100 kb of these loci (50-kb upstream and downstream of the lead SNP) were identified. The number of candidate genes in each locus range from 1 to 10, with an average of 3.65 (Supplementary Dataset 2). However, gene annotation in the maize genome is largely incomplete, and only about 1% of the genes have functional annotations based on mutant analyses; therefore, it is difficult to identify causal genes for a phenotype [48]. Whole genome gene expression is an easily measurable source of functional information. Several previous studies have used GWAS and RNA-seq data to detect causal genes for complex traits, such as root traits in rice [24], senescence, elemental concentrations, and oil biosynthesis in maize [20,48,49], and disease resistance in soybean [50]. We selected three long-root lines and three short-root lines for RNA-seq. Within GWAS-identified loci five genes showed differential expression between the long- and short-root lines. The functions of these genes need to be confirmed by mutant analyses or genetic transformation. 

## 5. Conclusions

In conclusion, we identified 34 QTLs associated with seedling root traits in a panel of 297 maize inbred lines by genome-wide association mapping. The expression levels of the genes located in GWAS-identified loci were assessed among long- and short-root lines by RNA-seq analysis. A total of five DEGs with potential causing variants were identified as strong candidate genes underlying seedling root development in maize. These genes can be further studied to help understand the genetic basis of root development and improve the root architecture.

## Figures and Tables

**Table 1:** Summary of the root and shoot traits and their measurements.

Abbreviation	Full Name	Unit	Measuring Method
PH	Plant height	cm	measured from the coleoptilar to yhe tip of the longest leaf
SPAD	Leaf chlorophyll concentrations	-	measured using a SPAD-502 PLUS chlorophyll (Minolta, Japan)
SDW	Shoot dry weight	10 mg	measured by electronic balance
ARD	Average root diameter	mm	measured by image analysis software (WinRhizo Pro 2009)
PRL	Primary root length	cm	measured by a ruler
SRL	Seminal roots length	cm	measured by a ruler
ASRL	Average seminal root length	cm	estimated by SRL and SRN
SRN	Seminal roots numbers	number	Count of the seminal roots
LRL	Lateral root length	cm	estimated by TRL, PRL and SRL
TRL	Total root length	cm	measured by image analysis software
RL005	L ≤ 0.5 (Root length between 0 and 0.5 mm in diameter)	cm	measured by image analysis software
RL0510	0.5 < L ≤ 1.0 (Root length between 0.5 mm and 1 mm in diameter)	cm	measured by image analysis software
RL1015	1.0 < L ≤ 1.5 (Root length greater than 1 mm in diameter)	cm	measured by image analysis software
RV	Root volume	cm^3^	measured by image analysis software
RA	Root surface area	cm^2^	measured by image analysis software
RDW	Root dry weight	10 mg	measured by electronic balance

**Table 2:** Phenotypic variation for root and shoot traits of 297 maize inbred lines.

Trait	Mean	SD	Min	Max	Fold Change	CV ^a^	ANOVA		
							MS (Genotype)	MS (Error)	F (Genotype)
PH	32.47	5.74	18.80	48.69	2.59	17.67%	63.36	7.70	8.23 ** ^b^
SPAD	28.47	3.86	17.05	38.15	2.24	13.55%	28.82	9.23	3.12 **
SDW	8.86	2.60	3.85	17.12	4.45	29.34%	12.34	2.38	5.18 **
ARD	0.44	0.06	0.33	0.66	2.00	14.16%	0.01	0.00	11.79 **
PRL	30.12	3.62	21.27	42.98	2.02	12.01%	25.68	4.11	6.24 **
SRL	67.08	22.71	6.60	141.00	21.36	33.85%	967.54	313.63	3.09 **
ASRL	23.95	4.40	11.71	34.83	2.97	18.39%	31.87	7.55	4.22 **
SRN	2.81	0.76	1.00	5.33	5.33	27.19%	1.09	0.60	1.83 **
LRL	265.88	111.46	34.35	687.12	20.00	41.92%	23267.84	1826.34	12.74 **
TRL	319.86	114.79	78.31	756.81	9.66	35.89%	24982.71	1914.59	13.05 **
RL005	236.78	96.32	42.79	599.84	14.02	40.68%	17497.93	1603.34	10.91 **
RL0510	75.60	26.60	16.03	143.26	8.94	35.18%	1372.08	173.74	7.90 **
RL1015	5.43	2.51	1.24	15.32	12.35	46.22%	12.23	2.39	5.12 **
RV	0.45	0.13	0.18	0.89	4.94	28.60%	296.39	30.66	9.67 **
RA	41.97	12.72	14.34	90.75	6.33	30.31%	0.03	0.00	8.23 **
RDW	9.15	2.43	2.40	15.75	6.56	26.52%	11.74	1.66	7.09 **

^a^ coefficient of variation. ^b^ indicates a significant effect was found by F test (*P* < 0.01).

**Table 3:** QTLs significantly associated with root traits detected by GWAS.

Trait	QTL	Lead SNP	Chr.	Pos. ^a^	Allele ^b^	−log_10_(*P*)	R^2^
ARD	*qARD1*	SNP_1_213315833	1	213315833	G/T	4.67	8.50
	*qARD3*	SNP_3_147397047	3	147397047	A/C	4.63	7.96
	*qARD4*	SNP_4_219099674	4	219099674	C/T	4.16	12.61
ASRL	*qASRL1*	SNP_1_121996211	1	121996211	C/T	4.35	9.49
	*qASRL3*	SNP_3_107899717	3	107899717	C/T	4.17	6.33
	*qASRL4*	SNP_4_185942913	4	185942913	A/T	4.89	7.01
	*qASRL10*	SNP_10_34244943	10	34244943	A/G	4.74	7.05
LRL	*qLRL2*	SNP_2_104416607	2	104416607	C/T	4.52	14.97
	*qLRL4*	SNP_4_168917747	4	168917747	C/T	5.39	9.27
PRL	*qPRL1*	SNP_1_120308712	1	120308712	C/T	4.30	6.34
	*qPRL10-1*	SNP_10_67350334	10	67350334	G/T	4.00	6.54
	*qPRL10-2*	SNP_10_68587247	10	68587247	A/G	5.38	9.29
RA	*qRA2-1*	SNP_2_104416607	2	104416607	C/T	4.45	14.65
	*qRA2-2*	SNP_2_184016997	2	184016997	C/T	4.05	6.99
	*qRA4*	SNP_4_168917747	4	168917747	C/T	4.57	7.58
RDW	*qRDW3*	SNP_3_156838240	3	156838240	C/T	4.70	8.29
	*qRDW4*	SNP_4_7135819	4	7135819	A/C	4.57	8.39
RL005	*qRL005_2*	SNP_2_104416607	2	104416607	C/T	4.86	15.87
	*qRL005_4*	SNP_4_33081784	4	33081784	C/T	4.48	12.29
	*qRL005_6*	SNP_6_63998692	6	63998692	A/G	4.97	10.66
RL0510	*qRL0510_1-1*	SNP_1_22179639	1	22179639	A/G	4.62	9.52
	*qRL0510_1-2*	SNP_1_217582572	1	217582572	G/T	4.81	10.10
	*qRL0510_2*	SNP_2_50742849	2	50742849	A/G	5.12	12.96
RL1015	*qRL0510_4*	SNP_4_34568124	4	34568124	G/T	4.05	11.43
	*qRL0510_6*	SNP_6_153696612	6	153696612	A/G	5.01	13.90
RV	*qRV2*	SNP_2_184016997	2	184016997	C/T	4.39	7.65
	*qRV6*	SNP_6_54506432	6	54506432	A/G	4.65	9.37
SPAD	*qSPAD5*	SNP_5_25046214	5	25046214	C/T	5.12	8.17
	*qSPAD8*	SNP_8_160371425	8	160371425	A/G	4.38	6.36
	*qSPAD9*	SNP_9_4832814	9	4832814	A/T	4.84	8.36
SRN	*qSRN2*	SNP_2_52559869	2	52559869	C/T	4.01	5.65
	*qSRN8*	SNP_8_19589120	8	19589120	C/T	5.42	9.39
TRL	*qTRL2*	SNP_2_104416607	2	104416607	C/T	4.64	15.22
	*qTRL4*	SNP_4_168917747	4	168917747	C/T	5.13	8.60

^a^ Position in base pairs for the lead SNP according to B73 RefGen_V3. ^b^ Underlined bases are the favorable alleles.

**Table 4:** Potential candidate genes identified by GWAS and RNA-Seq.

QTL	Candidate Genes	log_2_(FC) ^a^	*P*	SNP	Amino Acid Polymorphism	Annotation
*qARD3*	GRMZM2G138338	−1.38	0.032	39	8	Leucine-rich receptor-like protein kinase family protein
*qASRL4*	GRMZM2G174797	−1.44	0.00017	21	6	ELMO/CED-12 family protein
	GRMZM2G476902	3.74	0.00079	4	1	Armadillo/beta-catenin repeat family protein
*qLRL2/ qRA2-1/ qRL005_2/ qTRL2*	GRMZM2G397965	−2.78	0.0082	8	3	Vignain precursor/ (SAG12) senescence-associated gene 12
*qRDW3*	GRMZM2G031528	−2.48	0.00017	12	1	Heavy metal transport/detoxification superfamily protein

^a^ Log_2_ fold change between short-root lines and long-root lines.

## Supplementary Materials

The following are available online at https://www.mdpi.com/2073-4425/10/10/773/s1, Figure S1: Pearson correlation coefficients for root and shoot traits, Figure S2: Principal components analysis of traits in 297 inbred maize lines, Figure S3: Linkage disequilibrium decay across the whole genome in 297 inbred maize lines, Table S1: Linkage disequilibrium decay across the whole genome in 297 inbred maize lines, Dataset 1: Significant and suggestive SNPs detected by GWAS, Dataset 2: Identification of candidate genes in QTL regions detected by GWAS, Dataset 3: Gene differential expression between long- and short-root lines by RNA-seq analysis, Dataset 4: SNP and its annotation, Dataset 5: Statistics of the SNP in candidate genes.
