# Supplementary material for: Integrating GWAS and Gene Expression Analysis Identifies Candidate Genes for Root Morphology Traits in Maize at the Seedling Stage
Source: Genes (Basel). 2019 Oct 2;10(10):773. doi: 10.3390/genes10100773 (PMC6826382; doi:10.3390/genes10100773)
Supplement: Supplementary file 1 [file genes-10-00773-s001.zip › Supplementary Material-R1/Supplementary Material.docx]

Supplementary Materials

Journal Name: Genes

**Integrating GWAS and gene expression analysis identifies candidate genes for root morphology traits in maize at the seedling stage**

Houmiao Wang^#^, Jie Wei^#^, Pengcheng Li, Yunyun Wang, Zhenzhen Ge, Jiayi Qian, Yingying Fan, Jinran Ni, Yang Xu, Zefeng Yang*, Chenwu Xu*

Jiangsu Key Laboratory of Crop Genetics and Physiology/ Key Laboratory of Plant Functional Genomics of the Ministry of Education/ Jiangsu Key Laboratory of Crop Genomics and Molecular Breeding/Jiangsu Co-Innovation Center for Modern Production Technology of Grain Crops, Agricultural College of Yangzhou University, Yangzhou 225009, China

#: These authors contribute equally to this work.

^*^ Corresponding authors: Zefeng Yang

Email: zfyang@yzu.edu.cn

Chenwu Xu

Email: qtls@yzu.edu.cn

Tel: 86-0514-87979358

Fax: 86-0514-87996817

Table S1 Nutrient solution formulation for paper roll system

| Solution | Concentration (mM/L) |
| --- | --- |
| Ca(NO_3_)_2_ | 2 |
| K_2_SO_4_ | 0.75 |
| MgSO_4_ | 0.65 |
| KCl | 0.1 |
| KH_2_PO_4_ | 0.25 |
| H_3_BO_3_ | 1×10^-3^ |
| MnSO_4_ | 1×10^-3^ |
| CuSO_4_ | 1×10^-4^ |
| ZnSO_4_ | 1×10^-3^ |
| (NH_4_)_6_Mo_7_O_24_ | 5×10^-6^ |
| Fe-EDTA | 0.1 |


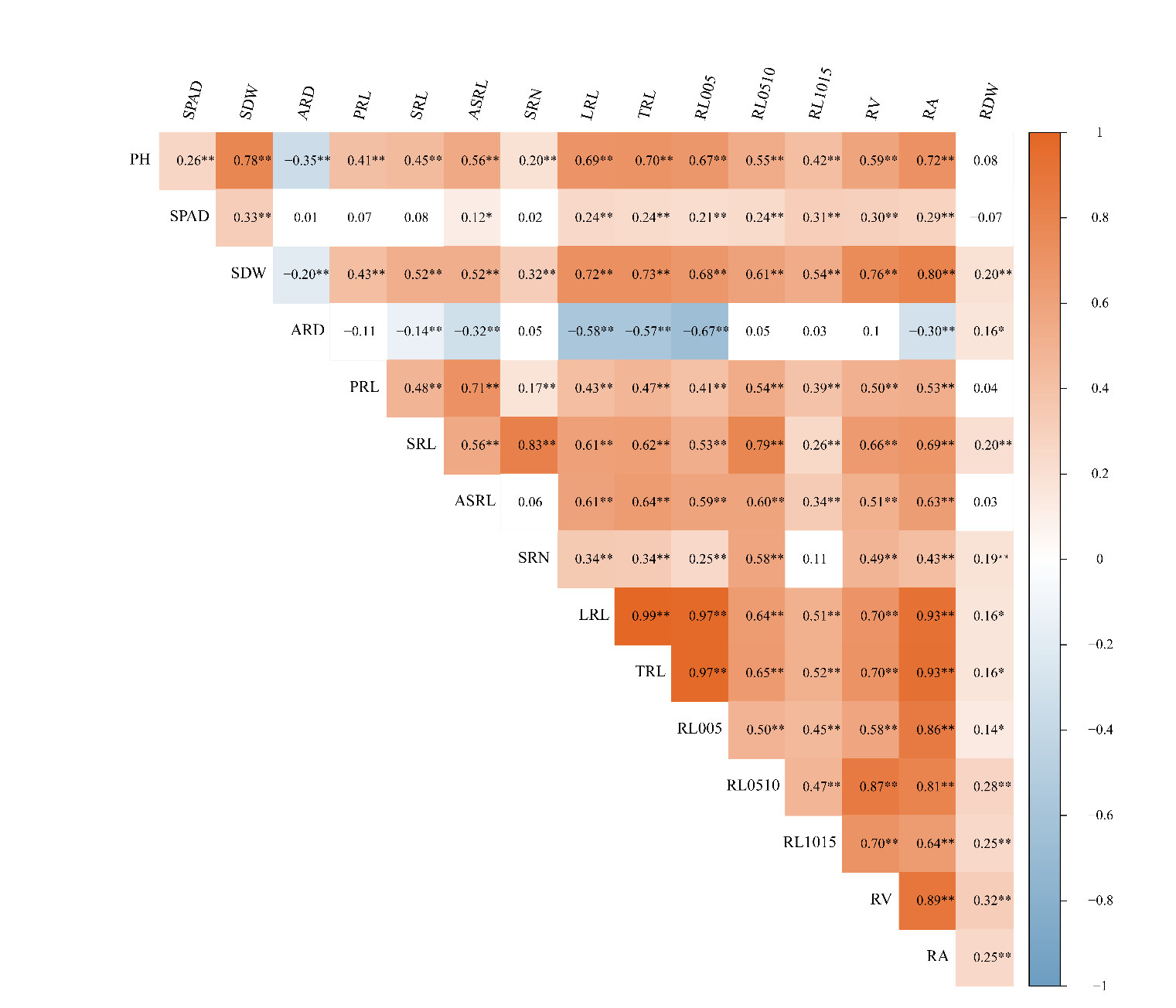


Figure S1 Pearson correlation coefficients for root and shoot traits.

(A)


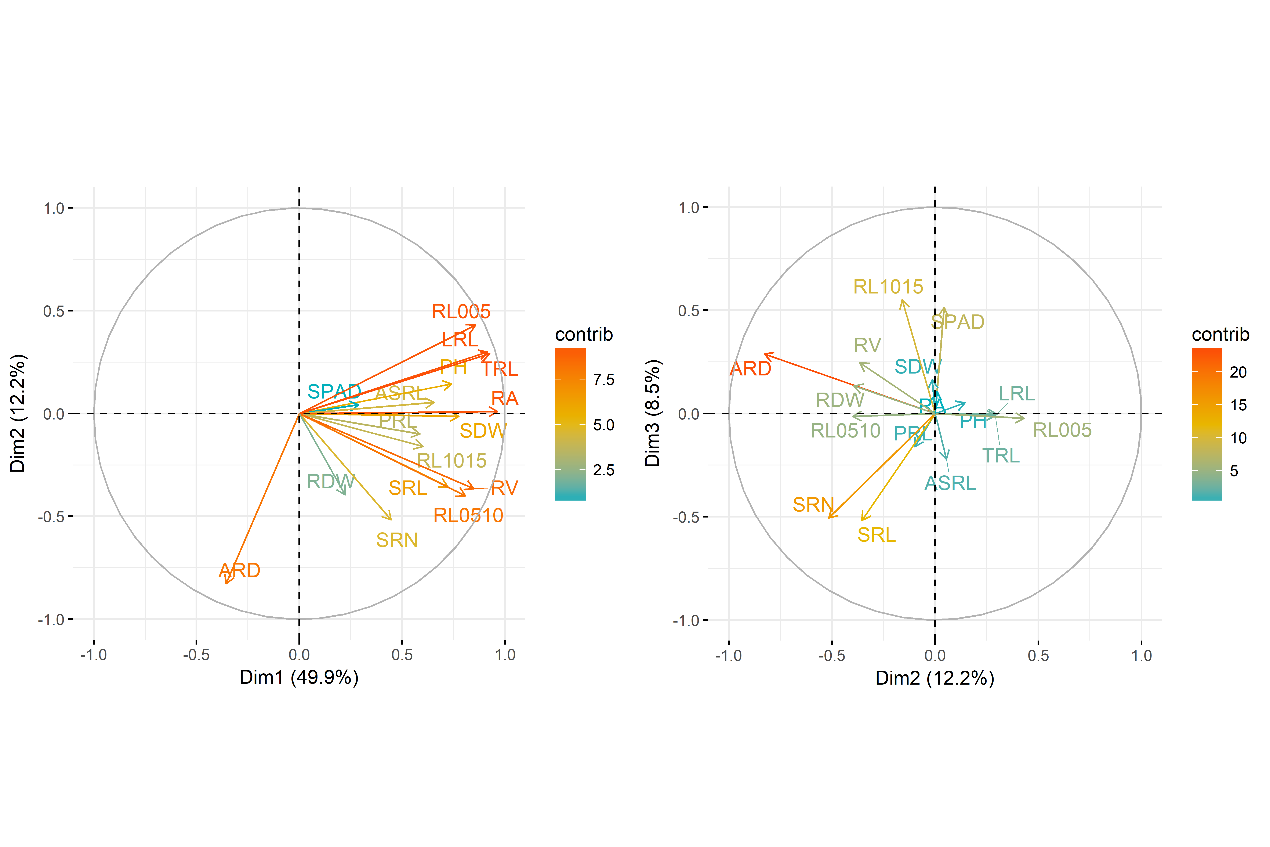


(B)


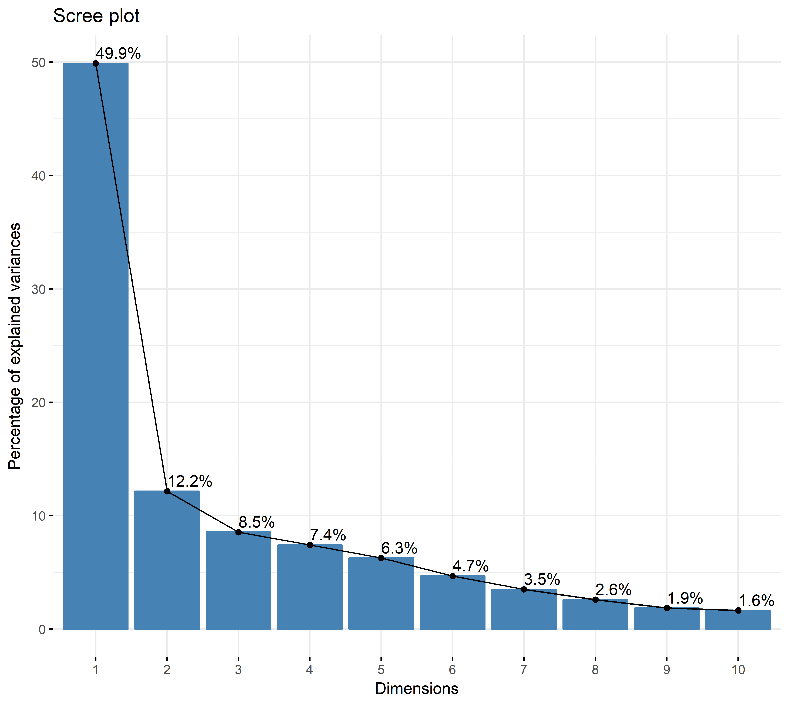


(C)
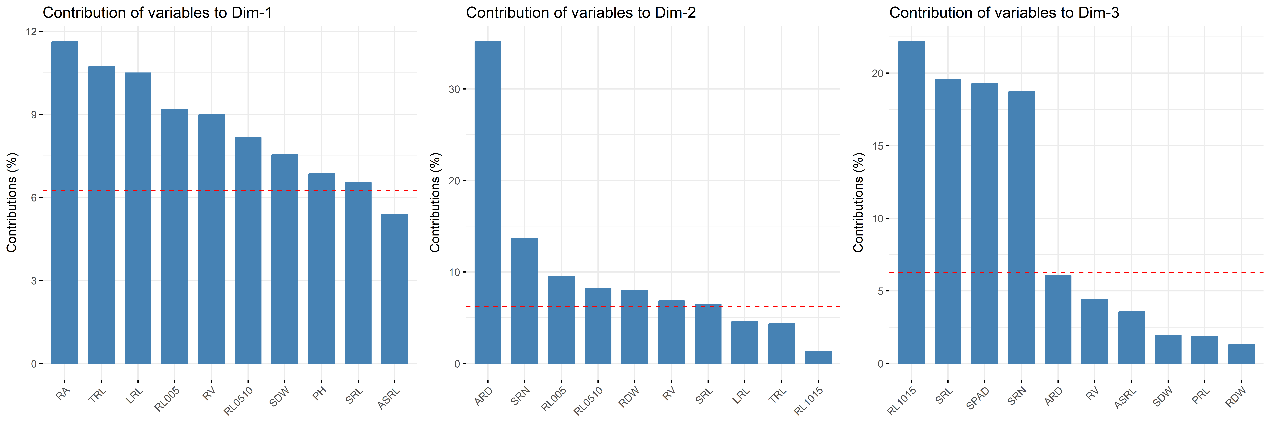


Figure S2 Principal components analysis of traits in 297 inbred maize lines. (A) The projection of 16 traits onto the first, second, and third dimensions. (B) Percentage of explained variances by the first ten dimensions. (C) Contribution of each trait to the dimensions.


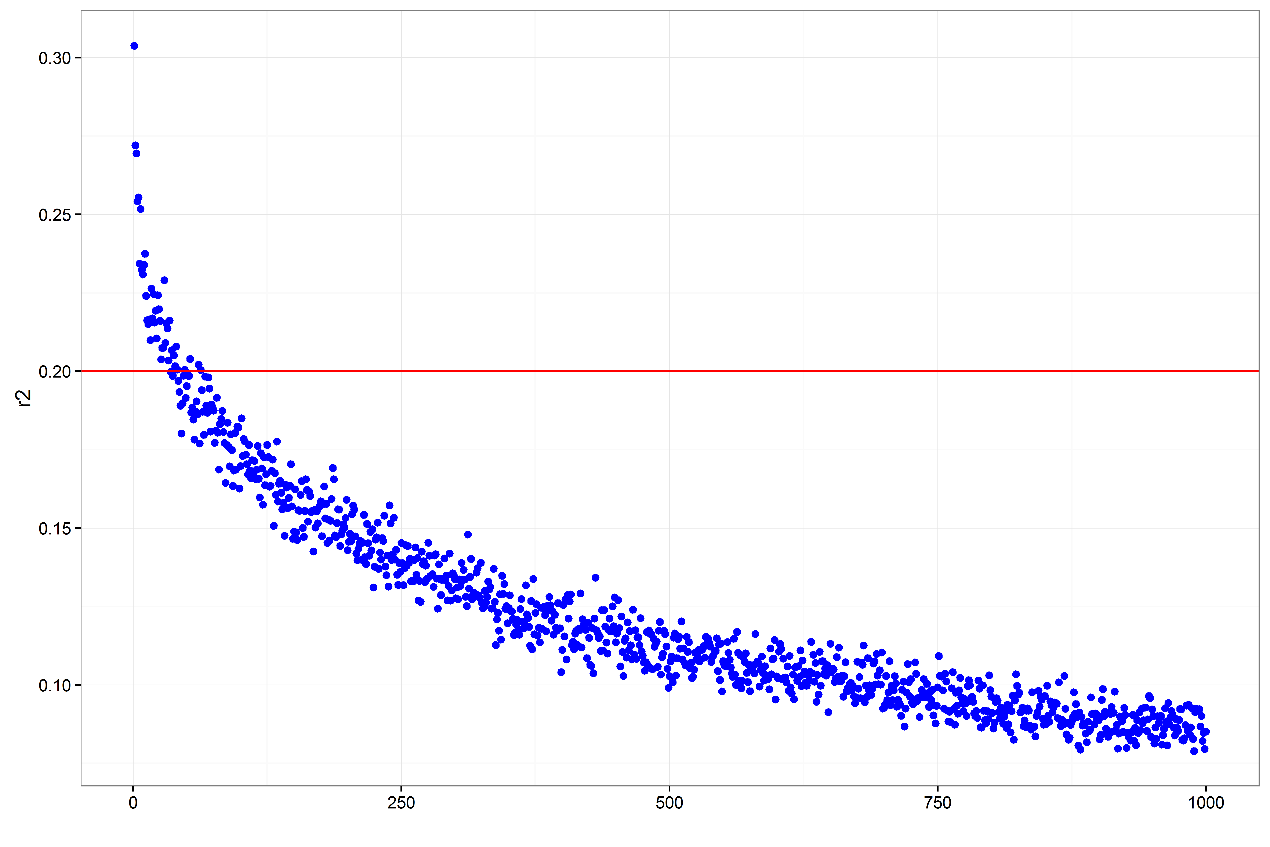


Figure S3 Linkage disequilibrium decay across the whole genome in 297 inbred maize lines.
